# Supplementary material for: MetaQTL: a package of new computational methods for the meta-analysis of QTL mapping experiments
Source: BMC Bioinformatics. 2007 Feb 8;8:49. doi: 10.1186/1471-2105-8-49 (PMC1808479; doi:10.1186/1471-2105-8-49)
Supplement: Additional File 7 — MetaQTL Package : jar file and tutorial. This Zip archive contains both the MetaQTL JAR file and the files of the tutorial. [file 1471-2105-8-49-S7.zip › org.inra.metaqtl/doc/index-files/index-8.html]

H-Index


|  |  |  |
| --- | --- | --- |
| **Overview** \| Package \| Class \| Use \| **Tree** \| **Deprecated** \| **Index** \| **Help** | |  |
| **PREV LETTER**   **NEXT LETTER** | **FRAMES**    **NO FRAMES**     **All Classes** |  |


A B C D E F G H I K L M N O P Q R S T U V W X Y Z 

---


## **H**

**hasChildren()** - Method in class org.thalia.bio.entity.OntologyTerm: **hasChildren()** - Method in interface org.thalia.bio.IBioOntologyTerm: Returns true if the term has children, false otherwise. **hasProba()** - Method in class org.metaqtl.graph.ChromLayer: **hasQTL()** - Method in class org.metaqtl.Chromosome: This method tests if the chromosome have a set of QTL defined. **haveSameFrame(int, int)** - Method in class org.metaqtl.CMarkerSequence: **HClustAlgorithm** - Class in org.metaqtl.algo: This class implements stantard agglomerative hierarchical clustering methods. **HClustAlgorithm()** - Constructor for class org.metaqtl.algo.HClustAlgorithm: **HEADER** - Static variable in class org.thalia.bio.entity.factory.OntologyTabFactory: **HEIGHT** - Static variable in class org.metaqtl.graph.GraphFactory: **height** - Variable in class org.metaqtl.TreeNode: The distance between the node and the root of the tree (if defined). **HORIZONTAL\_MODE** - Static variable in class org.metaqtl.QtlPartition

---


|  |  |  |
| --- | --- | --- |
| **Overview** \| Package \| Class \| Use \| **Tree** \| **Deprecated** \| **Index** \| **Help** | |  |
| **PREV LETTER**   **NEXT LETTER** | **FRAMES**    **NO FRAMES**     **All Classes** |  |


A B C D E F G H I K L M N O P Q R S T U V W X Y Z 

---
